# Evolutionary genomics: Insights from the invasive European starlings

**DOI:** 10.3389/fgene.2022.1010456

**Published:** 2023-01-04

**Authors:** Katarina C. Stuart, William B. Sherwin, Richard J. Edwards, Lee A Rollins

**Affiliations:** ^1^ Evolution & Ecology Research Centre, School of Biological, Earth and Environmental Sciences, UNSW Sydney, Sydney, NSW, Australia; ^2^ Evolution & Ecology Research Centre, School of Biotechnology and Biomolecular Sciences, UNSW Sydney, Sydney, NSW, Australia

**Keywords:** rapid adaptation, population genetics, *Sturnus vulgaris*, structural variants, plasticity, museum specimens

## Abstract

Two fundamental questions for evolutionary studies are the speed at which evolution occurs, and the way that this evolution may present itself within an organism’s genome. Evolutionary studies on invasive populations are poised to tackle some of these pressing questions, including understanding the mechanisms behind rapid adaptation, and how it facilitates population persistence within a novel environment. Investigation of these questions are assisted through recent developments in experimental, sequencing, and analytical protocols; in particular, the growing accessibility of next generation sequencing has enabled a broader range of taxa to be characterised. In this perspective, we discuss recent genetic findings within the invasive European starlings in Australia, and outline some critical next steps within this research system. Further, we use discoveries within this study system to guide discussion of pressing future research directions more generally within the fields of population and evolutionary genetics, including the use of historic specimens, phenotypic data, non-SNP genetic variants (e.g., structural variants), and pan-genomes. In particular, we emphasise the need for exploratory genomics studies across a range of invasive taxa so we can begin understanding broad mechanisms that underpin rapid adaptation in these systems. Understanding how genetic diversity arises and is maintained in a population, and how this contributes to adaptability, requires a deep understanding of how evolution functions at the molecular level, and is of fundamental importance for the future studies and preservation of biodiversity across the globe.

## Introduction

Evolutionary theory states that the immense diversity existing on this planet does so through a complex combination of factors. These factors include genetics, epigenetics, and plasticity, and it is the interplay of these processes that allow species to evolve within our increasingly changing world (Goudie, 2018). We are ever gaining an appreciation for both the speed at which evolution occurs (Prentis et al., 2008), and the role humanity plays in shaping it (Sih et al., 2011). Thus there is much interest in the central role genetic variation plays in facilitating a population’s evolutionary potential, so that we may better understand why some persist and others perish.

Population and evolutionary genomics play pivotal roles in answering these questions. Technical advances have given rise to cheap “reduced representation” data (subsampling genomic variation) and the growing feasibility of whole genome resequencing (WGS) for non-model organisms. Population genetics can now move beyond the characterisation of broad genetic patterns to uncover evolutionary important genetic variants at a resolution previously inaccessible to non-model organism studies (Hendricks et al., 2018; Hohenlohe et al., 2019, 2021). Examining genetic patterns across, for example, environmental (e.g., Gugger et al., 2017) or morphological (e.g., Nannan et al., 2022) landscapes, enables us to develop hypothesis regarding the drivers of a population or species’ genetics.

Studies in invasive species genomics are fundamental to these efforts. By examining how invasive populations’ genetic diversity is shaped by novel selection regimes, we obtain insight into the molecular patterns underpinning evolution. Invasive species, by nature, are successful following genetic bottlenecks (i.e., the large reduction in effective population size that occurs during translocation), and provide an avenue for understanding what aspects of genetic diversity (e.g., transposable elements; Stapley et al., 2015, or specific chromosomes; Meisel & Connallon 2013; Waters et al., 2021) contribute to adaptation under a new selection regime. Through such studies, we begin to appreciate the complex nature of genetic variation and how this may facilitate rapid local adaptation, and thereby species persistence, in response to an altered environment under a future of climate change (Razgour et al., 2019; Waldvogel et al., 2020). In this perspective, we discuss recent discoveries in genetics of the invasive European starlings within Australia, and use these studies to prompt interesting avenues for further research more broadly across the fields of evolutionary and population genetics.

## Perspectives from the study of the European starling

**FIGURE 1:**
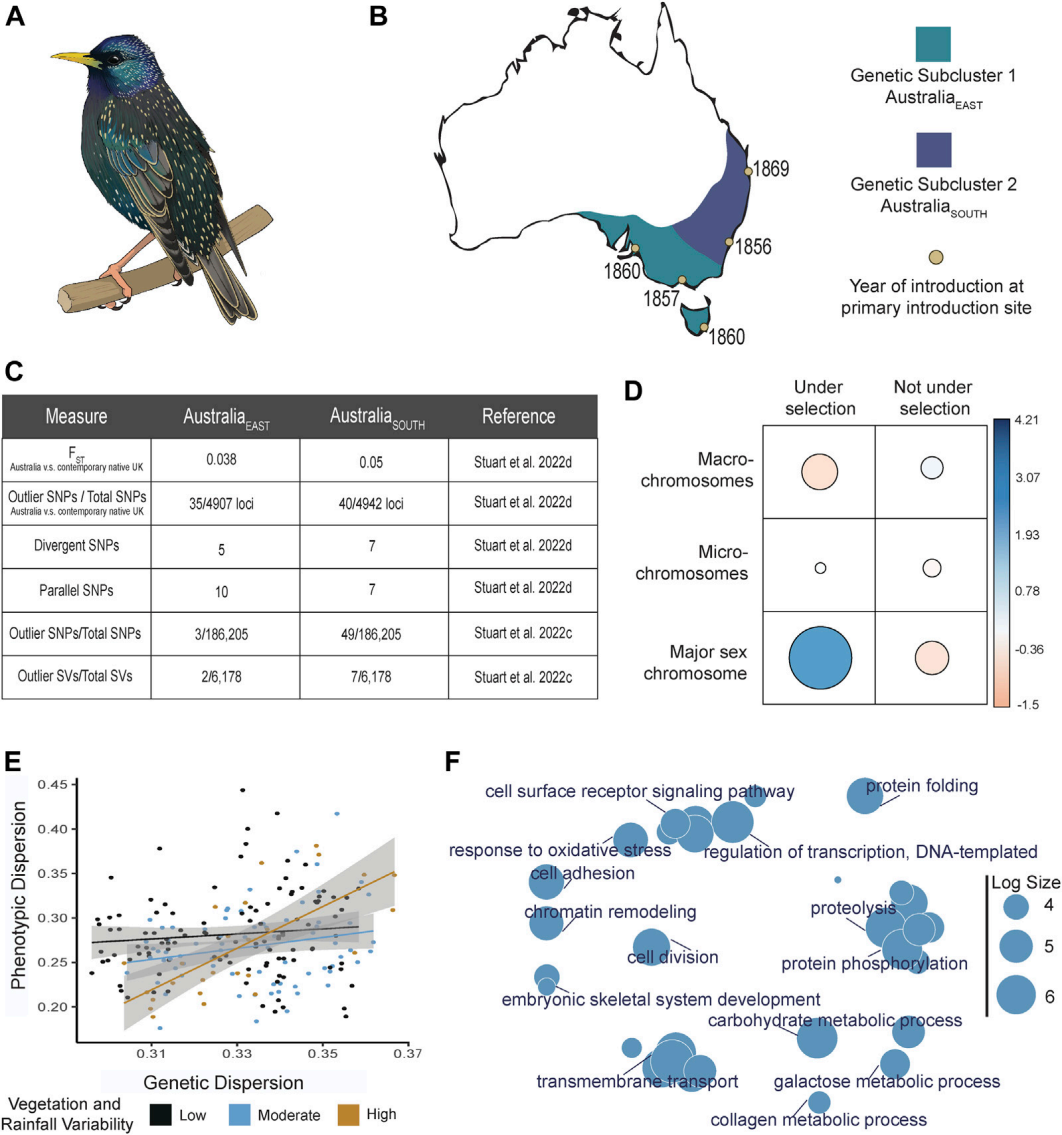
Summary diagram of evolutionary trends for *Sturnus vulgaris* (*S. vulgaris*) within the invasive Australian range. Panel **(A)** depicts an artist’s image of a male *S. vulgaris* in breeding season. Panel **(B)** depicts the Australian range of *S. vulgaris* (approximately based on eBird data retrieved 2018), with approximate genetic sub-structuring indicated in purple (Australia_EAST_) and blue (Australia_SOUTH_), and with introduction sites indicated (yellow circles) next to the year of first introduction. Panel **(C)** depicts differences in genetic differentiation between the two Australian subpopulations (Australia_EAST_ and Australia_SOUTH_) and the native range. F_ST_ values were obtained from comparisons to Newcastle, UK, from Stuart et al. (2022d). Panel **(D)** depicts a subset of the chi-squared results that assessed the occurrence of putative outliers and non-outlier SNPs across macro-, micro-, and major sex (Z) chromosome from Stuart et al. (2022d). The table visualises the Pearson residuals, where the circle area is proportional to the amount of the cell contribution, positive residuals (indicating a positive correlation) are in blue, and negative residuals (indicating a negative correlation) are in orange. Panel **(E)** depicts the positive interaction between phenotypic and genetic dispersion, which is positively affected by the level of ground cover vegetation and annual precipitation variation, data pulled from Stuart et al. (2022a). Panel **(F)** depicts a revigo (Supek et al., 2011) gene ontology term summary plot of all coding regions that were identified as significant across loci identified in studies on the Australian starling population: all statistically-outlier and environmentally-associated loci (Stuart et al., 2021); all divergent and parallel loci (Stuart et al., 2022d); all statistically outlier SNPs and SVs identified by Bayescan v2.1 (Foll and Gaggiotti, 2008) (Stuart et al., 2022c); all morphologically-associated loci that were also under selection (Stuart et al., 2022a). Log size is indicative of the frequency of the GO terms.

Of the 17,000 species that have been labelled as invasive (Seebens et al., 2017), the European starling (*Sturnus vulgaris*) (Figure 1A) is a standout. As one of the only birds on the IUCN’s top 100 worst invasive species (Lowe et al., 2000), the starling, despite suffering dramatic population declines within its native palearctic range (Bowler et al., 2019), has colonised every other continent, barring Antarctica. The repeated and well documented introductions, combined with extensive natural history, genetic, and other biologically relevant data (e.g., environmental, phenotypic), has and will continue to yield many exciting discoveries in molecular evolution. With recent publication of several genetic studies on the invasive starling populations (focused primarily on Australia) comes an opportunity to synthesise key results, and propose broad hypothesis regarding the nature of their rapid evolution in native and introduced populations.

### Complex introduction histories in human-mediated populations impact selection analysis

The Australian starling invasion has a well-documented, but complex, introduction history, with multiple geographically dispersed introduction points that are separated in some instances by thousands of kilometres (Figure 1B). Starling population genetics has demonstrated that while invasive populations provide a valuable resource for evolutionary studies, we must also acknowledge the challenges of separating selective and neutral evolutionary processes for populations with complex introduction histories. Demographic processes during range expansion may create false signals of selection in genetic data, or may even mask legitimate signals (Stuart et al., 2021). Further, a continuous invasive range may have resulted from numerous separate introductions that themselves were exposed to different selection regimes (Figure 1C), either at the introduction site or along environmental clines during range expansion (e.g., Stuart et al., 2022a). This selection co-occurs alongside source population differences and stochastic demographic effects (e.g., drift), hence it may be impossible to separate some genuine signals of selection from neutral genetic processes within some invasions. Strong subpopulation structure and recent range expansion may confound local signatures of adaptation, and therefore analytical approaches should take this into account (e.g., Stuart et al., 2022d; 2022c). The impact of separate introduction sites or range expansions on population-wide genetic variation is something that may need to be considered not just within invasive populations, but during genomic studies on non-invasive populations (e.g., Drury et al., 2017; Drinan et al., 2018; Pertoldi et al., 2021), and species reintroductions (e.g., Kaulfuss & Reisch 2017; Dincă et al., 2018; Mims et al., 2019).

### Patterns of evolutionary change within the *Sturnus vulgaris* genome

Exploration of starling populations identified several broad trends that characterize patterns of genetic change in this species. First, comparing invasive populations on separate continents (Hofmeister et al., 2021), or distinct subpopulations within a single invasive range (Stuart et al., 2022d), reveals that separate populations may experience parallel selection across geographically isolated regions. Further, selection (parallel or divergent) is not restricted to translocated invasive range, but may also occur within the native range post divergence (Stuart et al., 2022d). Considering this, it is vital that evolutionary studies comparing putatively adaptive differences between populations do not assume either the origin of parallel signatures of selection, or that genetic divergence has occurred only or even primarily within invasive ranges, because this may limit interpretation of results.

Second, selection (parallel or divergent) within starling populations often occurs at sites with moderate allele frequencies (Stuart et al., 2022d), and levels of balancing selection (selection that maintains multiple allele variants within a population) vary across different types of genetic variants (Stuart et al., 2022c). This supports the theory that balancing selection within native populations has the potential to maintain evolutionarily important alleles (Hedrick, 2007) that may undergo directional selection within novel ranges, assisting an organisms’ ability to rapidly adapt to new selection regimes (Stern and Lee, 2020). Understanding the mechanisms maintaining standing genetic variation within populations, particularly for functional variants of different types (e.g., single nucleotide polymorphisms—SNPs, structural variants—SVs) has important implications for conservation genetics (e.g., Whiteley et al., 2015; Lai et al., 2019) and thus is an extremely pressing research direction.

Third, examining the location of putative sites under selection across the starling’s genome reveals a larger number of genomic sites than expected under selection in the major sex (Z) chromosome (Stuart et al., 2022d, Figure 1D). These results concur with existing research on the role that sex chromosomes play in rapid evolution (Meisel & Connallon 2013), and is in alignment with results that highlight the importance of the Z chromosome specifically within avian divergence and speciation (Campagna et al., 2017).

Lastly, assessing genetic alongside environmental and morphological data indicates that genetic variation is positively correlated with phenotypic variation, and this relationship becomes more pronounced under a temporally variable environment (high vegetation and rainfall variability; Stuart et al., 2022a, Figure 1E). While the exact details of these results (e.g., driving climate factors) are system specific, they demonstrate the importance of assessing variation across data types to better understand drivers of phenotypic plasticity (which may in the future be aided by the inclusion of epigenome data in plasticity studies). Importantly, because climate change increases environmental variability (Thornton et al., 2014), such studies on invasive populations will allow us to understand how genetics and environments, and their interactions, shape rapid adaptive change under novel selection regimes.

### Signals of selection correlated with environmental variables

Strong genetic differences between the invasive Australian and North American starling populations (Hofmeister et al., 2021; Stuart et al., 2021) showcase the flexible ecology of this globally invasive species, demonstrating the difficulties in predicting potential species’ ranges (Whitney and Gabler 2008). A focal research direction within recent starling genomic studies sought to understand how the hotter, more arid Australian environment shaped the introduced starling population. Within the two major Australian subpopulations, the southern subpopulation cluster is more divergent from the native range than the eastern one (Stuart et al., 2022c, 2022d, key results summarised in Figure 1C). While these patterns may have resulted from founder genetics or stochastic processes, they may also be indicative of different selection regimes experienced within subpopulations. There are strong bottleneck effects at the Australian western-most range-edge, along with high proportions of private alleles for both SNPs and SVs (Rollins et al., 2011; Stuart et al., 2021; 2022c). Likely, this range-edge differentiation (previously attributed to an extreme genetic bottleneck) resulted from accidental introduction/s from elsewhere in the starlings’ native range.

These studies have flagged many coding regions under putative selection within the invasive Australian range (Figure 1F). These contain genes covering a range of putatively adaptive functions (e.g., immune response, beak morphology), including a diverse range of novel seeking behaviour associated genes (Stuart et al., 2021; Stuart et al., 2022c, Stuart et al., 2022d, but see Rollins et al., 2015). Starlings’ intelligence and behavior has been the focus of much research (Mueller et al., 2014; Nettle et al., 2015; Van Berkel et al., 2018), and with research linking invasion success to behavioral flexibility (Sol et al., 2002), follow-up studies on these candidate genes will shed light on rapid adaptation in novel seeking behavior within starlings.

Rapid adaptation, occasionally in response to environment, has impacted starling phenotype. Analysis of morphology-associated loci under putative selection indicated that climate extremes are more important than means in explaining morphological patterns (Stuart et al., 2022a), echoing findings within other species (e.g., Vasseur et al., 2014; Gardner et al., 2017). Further, climate correlates of genetic patterns were highly varied across overall genetic patterns, morphology-associated genetic patterns, and phenotype and genetic variance patterns. Collectively, these results demonstrate how important it is to examine different components of a genetic landscape to best understand what is driving adaptive change.

## Areas for future expansion in population and evolutionary genomics

Synthesis of recent findings in starling genetics has identified important growing themes and promising future directions in population and evolutionary genomics research. These avenues will provide invaluable insights into the fundamentals of genomic evolution, with application in both invasive and non-invasive systems.

### The utility of museum collections

Sequencing historical samples from museum collections facilitates a range of potential new genomic projects to, for example, track temporal changes in allelic landscapes, or conduct studies into now-extinct lineages. Analysis alongside contemporary samples in both our study and others is promising (e.g., Ewart et al., 2019; Parejo et al., 2020; Yao et al., 2020). Across many institutions, research is ongoing into different aspects of museum sample utility, including extraction methods (Tsai et al., 2020; Hahn et al., 2022), DNA recovery from specimen ethanol (Jeunen et al., 2021), and improving wet lab—bioinformatic hybrid approaches (Bernstein and Ruane, 2022) to maximise data from rare and degraded specimens (Raxworthy and Smith, 2021). Assessing, for example, morphological and genetic change over time, may be used to better appreciate how industrialization, human land use, and climate change has affected a wide range of species. Conducting such studies across both successful invasives and vulnerable geographically-isolated endemics would facilitate deeper understanding of how population expansion (or decline) tracks with underlying genetic diversity and anthropogenic effects.

### The need for collection of phenotypic data alongside genetics

While phenotypic data is often well integrated into genetic studies in agricultural species (e.g., Reynolds et al., 2021), managed native species (e.g., Álvarez-Varas et al., 2021), or even plant invasions (e.g., Bhattarai et al., 2017), there is a general lack of such data in invasive animal studies. Even when phenotype data are collected, sampling wild populations means that some data remains unknown (e.g., pedigree information) and many traditional analytical techniques (e.g., heritability analysis) may require unobtainably large sample sizes. And while museum collections may enable easy morphological data collection, such collection efforts must contend with preservation method related shrinkage (Maayan et al., 2022). However, these difficulties should be and are being overcome (e.g., Hedrick et al., 2018), because pairing phenotype data with the underlying genetic data is vital for understanding the role plasticity plays in invasions (e.g., Santi et al., 2020), as well as identifying critical genome regions that may facilitate rapid phenotypic adaptation (e.g., Wu et al., 2019). Understanding heritability and plasticity is necessary for long-term modelling of invasive populations, and to explore the limits of a species’ ability to adapt to shifting selection regimes.

### Beyond the genome: A multi-omics approach to adaptation

General consideration of phenotypic plasticity is important for establishing the limits of genetic (evolved) contributions to adaptation. However, phenotypic plasticity itself can have different underlying causes and mechanisms. Deeper understanding the role of plasticity in shaping a populations’ adaptive potential requires expanding omics data collection beyond just the genome. Co-analysing genetic and phenotypic data will be greatly aided by the inclusion of, for example, transcriptome, proteome, or epigenome data (Layton and Bradbury, 2022). These complimentary data sets will provide vital information about the biological processes that link the underlying heritable DNA of an organism to its resulting phenotype. Exciting new techniques are allowing us to obtain this information from previously inaccessible samples (e.g., Hahn et al., 2020; Rubi et al., 2020). Analysing multi-omics data sets across temporal and spatial landscapes will shed light onto the complex interactions between the heritable genetic, heritable epigenetic, and non-heritable plastic elements that collectively contribute to a populations’ adaptive potential.

### Putatively adaptive loci

Evolutionary genomic studies often produce a list of genetic sites flagged as under putative selection or associated with phenotypic or environmental data. While generating a shortlist of biologically interesting variants is the first of many steps towards biologically validating these results, knowing whether a variant has an adaptive advantage (or disadvantage) may require additional data (e.g., RNA expression or phenotype data), or even “evolve and resequence” studies under one or more standard environmental conditions (Schlötterer et al., 2015). It is vital that we go beyond compilation of outlier loci and begin using this often end-result as a stepping stone to further scientific inquiry. Analytical advancements, such as Alphafold (Jumper et al., 2021) and Variant Effect Predictor (McLaren et al., 2016), will help evolutionary biologists ask more of their data, and reciprocally increase our understanding of science across research fields. We may begin to use flagged outlier loci across a range of species to examine broad questions about, for example, genomic positioning of selective loci, protein sequence change trends, and 3D protein structure impacts. Additionally, for well-studied systems like the starling, it may be worthwhile to curate species-specific databases of variants of interest, as often genes of import are accessible only by manually trawling through literature, and non-coding flagged loci are even more inaccessible (if recorded at all). Compiling outlier variant data across studies is vital if we want to develop inter-specific perspectives. Ultimately, through clear scientific reporting of the results of individual exploratory studies we may begin to collectively resolve broad trends in molecular evolution.

### Genomic meta-analysis and whole genome resequencing

**FIGURE 2:**
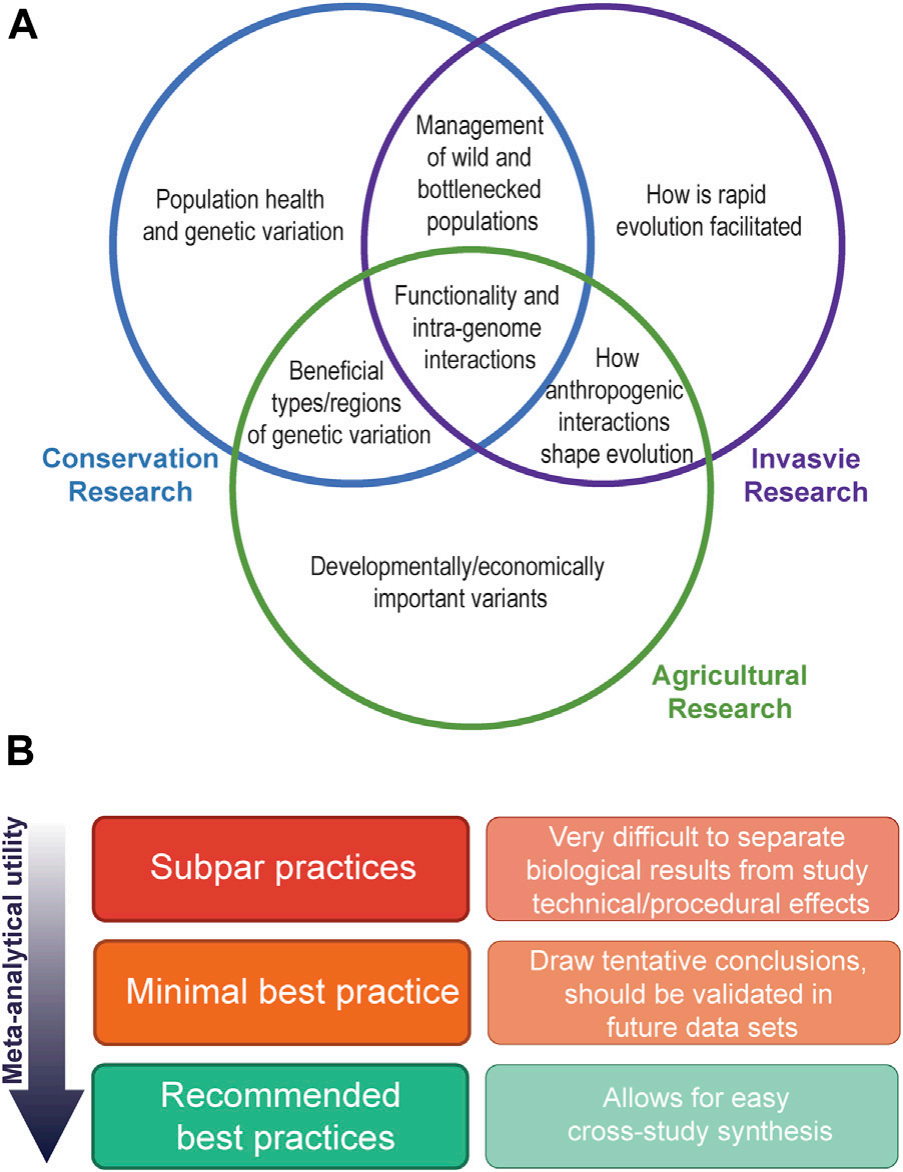
Visualisation of the possible outcomes and data requirements of cross study synthesis in evolutionary and population genetics. Panel **(A)** depicts common research outcomes from studies of invasive, conservation, and agricultural population and evolutionary genomics research, and their overlap. Panel **(B)** depicts the need for highly curated and reproducible datasets to enable confident cross-study comparisons. Best practices will often be specific to the variant types being called and the research setting they are being conducted in (e.g., Olson et al., 2015; Cameron et al., 2019; Koboldt 2020), and will often be limited by funding and resource availability. In general, minimal best practices should be informed by both the technical limitations of the sequence technology used (e.g., quality checks of the right type and stringency), as well as biologically informed decision making (e.g., accounting for population structuring when conducting downstream analysis such as outlier detection).

As we acknowledge the utility in cross-study comparisons of variants of interest, so too must we acknowledge the lack of these within the field of evolutionary and population genomics. Such approaches can start more modestly, with comparisons across similar species (for example, comparison between the invasive sturnids, the starling and the common myna *Acridotheres tristis*) to yield insights into the repeatability of adaptive change in invasive taxa. While the lack of comparative studies is simply because the field is in its relative infancy, we are quickly moving into an age where enough studies have now been published for us to begin employing meta-analytical approaches to conduct formal inter -population or -specific comparisons. This will allow us to begin confirming whether results from a singular study are an isolated phenomenon within a particular system, or are broadly applicable over many. Comparing patterns of genomic change across a broad range of invasive species is of vital importance to invasion population genomics to answer questions about the importance of different genomic variants and the role they play in rapid adaptation and species persistence. Further, comparisons between invasion genomics and the fields of conservation and agricultural genomics also promise to yield interesting answers (Figure 2A).

Technology continues to shape the nature of the questions that can be asked and reveals the importance of previously understudied types of variation. WGS captures genome-wide genetic information that improves on reduced representation sequencing approaches, as it does not rely on subsampling species-specific genomic loci. This provides a more uniform starting point for further analysis and more long-term utility, provided careful scientific reporting and best practices are followed (Figure 2B). Further, WGS has broadened investigation of non-SNP genetic variants, such as SVs and transposable elements, which are of growing interest to the evolution and population genomics community. These investigations are increasingly served by shifts to “third generation” technologies and the promise of affordable, high accuracy long-read sequencing.

### Pan-genomes

The incorporation of published data into cross study comparisons is greatly aided by a high-quality species genome. While putative chromosomes may assembled using syntenic approaches (e.g., Stuart et al., 2022b), completely de-novo assemblies with long-range scaffolding data incorporated are necessary for confidence in, for example, structural rearrangements. However, even these platinum standard genomes are superseded by pan-genomes, which is a genome map that attempts to capture species-wide genetic diversity and structure (Vernikos et al., 2015; Sherman and Salzberg, 2020). Pan-genomes will help to alleviate reference biases introduced by mapping many, possibly quite genetical distinct individuals of a species to a reference genome that only represents the genetic structure of one individual (and thus may fail to provide an adequate map for more divergent regions within the resequenced individuals), increasing data integrity and hence utility (Figure 2B). While the creation of such a resource is currently not financially feasible for most studies, existing genomes can be incorporated into future pan-genomes. To fully characterize genetic divergence between populations, particularly in hard to map genomic areas (such as regions with high repeat content), pan-genomes are essential.

## Conclusion

The study of invasive systems have and will continue to yield many important discoveries within the fields of population and evolutionary genomics. Characterising rapid adaptation from the molecular to the macro level within invasive populations enables scientists to appreciate how genetic variation interacts with a variety of selection processes to allow species to evolve. Within these studies, starlings present a valuable system for understanding evolution, providing opportunity to investigate everything from subtle morphological shifts within a region, to observing broad patterns of parallel change across the globe. The research conducted on this system demonstrates the complex nature of standing genetic diversity and the means through which it facilitates adaptation. From these results we also see some promising directions of future study within the field of evolutionary and population genomics. Collectively pursuing these directions using this system and that of other invaders will facilitate a deeper appreciation of how evolution functions at the molecular level. Ultimately, through deep studies across a broad range of taxa, we may learn to precisely explain and predict patterns of evolution, to protect precious biodiversity.
